# Potential of some explants for callus induction and plantlet regeneration in *Solanum lycopersicum* L. under treatment of different plant growth regulators

**DOI:** 10.5114/bta.2024.141803

**Published:** 2024-09-30

**Authors:** Anjana Kumari, Avinash K. Nagpal, Jatinder K. Katnoria

**Affiliations:** Department of Botanical and Environmental Sciences, Guru Nanak Dev Universtiy, Amritsar, Punjab, India

**Keywords:** callus, PGRs, regeneration, explants

## Abstract

Plant growth regulators (PGRs) control signaling networks and developmental processes involved in plant responses to various biotic and abiotic stresses, making it crucial to study PGRs *in vitro*. The protocol for micropropagation of *Solanum lycopersicum* L., following callus induction and regeneration through explants such as internodal segments, leaves, and nodal segments, was established during the present study. Explants were inoculated on Murashige and Skoog (MS) medium supplemented with different plant growth regulators like BA (6-benzylaminopurine), 2,4-D (2,4-dichlorophenoxyacetic acid), BA + 2,4-D, IAA (Indoleacetic acid), IBA (Indolebutyric acid), and NAA (Naphthaleneacetic acid). It was observed that among all explants, the nodal segment showed maximum callus induction (83.33%) and multiplication (86.67%) at 0.25 mg/l of 2,4-D; the highest shoot number (3.33) at 0.5 mg/l of IAA + 0.5 mg/l of BA; the greatest shoot length (7.57 cm) at 0.75 mg/l of BA; root induction (80.95%), root number (21.88), and root length (10.62 cm) at 1 mg/l of IAA. Additionally, the maximum fresh weight (2.448 g) was observed at 0.5 mg/l of BA, while the highest dry weight (0.172 g) and dry matter content (14.25%) were noted at 1 mg/l of BA + 1 mg/l of 2,4-D for the internodal segment. Results of the present study revealed that among different explants, the best response was given by nodal segments, followed by internodal segments. Among the different PGRs, 2,4-D resulted in the highest callus induction and multiplication percentage.

## Introduction

*Solanum lycopersicum* L. (tomato) is a popular seasonal vegetable crop cultivated worldwide and a Solanaceae family member (Foolad, 2007; Kumar et al., 2022). This vegetable crop has gained attention in the last decades due to its low sugar content and the presence of lycopene, a medicinal constituent that possesses antioxidative and anticancer properties (Wu et al., 2011; Raiola et al., 2014). *S. lycopersicum* is recognized as a vegetable with many culinary applications and as a nutrient-rich superfood offering a broad spectrum of benefits to the human body. It is a rich, fibrous fruit that contains large quantities of L-ascorbic acid, choline, and potassium, and its consumption is documented as healthy for the human heart (Kim et al., 2011).

Due to its many medicinal properties, *S. lycopersicum* is considered the fourth most prevalent fresh vegetable, with potato standing first, lettuce second, and onion third (Canene-Adams et al., 2005). For *in vitro* studies, the tomato is an auspicious vegetable crop because of its small chromosome number (2*n* = 24) and the wide available information on its inheritance. Three cultivars of tomato were studied to optimize the protocol for callus induction, proliferation, and regeneration, and it was reported that Riogarande had the best response in MS medium supplemented with 1.5 mg/l of 2-isopentenyl adenine (IP) and 0.5 mg/l indole-3-acetic acid (IAA) (Chaudhry et al., 2007). In other studies, shoot apex, nodal, and internodal segments, roots, and cotyledons were used as explants for callus induction and regeneration of tomatoes (Jatoi et al., 2001; Sherkar et al., 2014; Yaroshko et al., 2023).

The tissue culture technique is a useful tool to improve the productivity of vegetable or food crops through the availability of healthy plant material (Bhatia et al., 2004; Vargas and Alego, 2018) as well as to develop certain cultivars in limited or short-span periods (Taji et al., 2002; Hussain et al., 2013; Brar and Khush, 2021). This technique is also used to raise valuable plant species that are difficult to regenerate using traditional methods, as well as for endangered species that need conservation. Additionally, the tissue culture technique has been successfully used for the growth and production of biotic stress (pathogen) free plants. Unlike traditional methods, under *in vitro* conditions, plants require minimal time and space for the production of millions of plantlets (Ashrafzadeh and Leung, 2015). Various protocols for *in vitro* production of several plants have been developed so far. The first true tissue culture plant was obtained by raising Acer pseudoplatanus using its cambial tissue (Gautiieret, 1935).

Plant regeneration under *in vitro* conditions has been observed to depend on various factors such as the composition of the growth medium, including the basal medium, plant growth regulators, and gelling agent; and physical factors such as temperature, relative humidity, intensity, duration, and quality of light (Reed, 1998; Haque et al., 2022). The success of plant tissue culture also depends on the effective establishment of a culture system, which consists of a capable genotype and efficient explant material with optimal conditions (Plana et al., 2005; Tiwari et al., 2021). Various hormonal combinations with BA, 2,4-D, IAA, and Kinetin (KN) have been successfully used to induce callus and regeneration in many plants such as *Nicotiana tabacum*, *Salvia moorcroftiana*, and Glycine max (Lang et al., 2020; Zarad et al., 2021; Bano et al., 2022; Muthu et al., 2023). However, very few reports are available in the literature for callus induction and multiplication of *S. lycopersicum*, which is a very valuable vegetable crop in terms of its nutritive and medicinal importance.

Keeping this in mind, the present study was planned to explore the potential of *S. lycopersicum* for callogenesis and regeneration from three explants: leaves, nodal, and internodal segments, in the presence of different plant growth hormones.

## Materials and methods

### Explant collection

Seeds of *S. lycopersicum* L. variety (Punjab Kesar Cherry) collected from Punjab Agriculture University, Ludhiana, Punjab (India) were sown in soil under greenhouse conditions at “Mata Kaulan” Botanical Garden, Guru Nanak Dev University, Amritsar, Punjab (India) to raise plants. Different explants (leaves, nodal segments, and internodal segments) were taken from twenty-day-old healthy plants and were used for callus induction and multiplication.

### Preparation of plant growth hormone solution

Chemicals (HCl and ethanol) used in the present experiments were procured from Qualigens (Mumbai, India), whereas plant growth regulators from Sigma-Aldrich were used. One hundred milligrams of plant growth hormone powder was dissolved in 2–3 drops of HCl or 70% ethanol, and double-distilled water was added to make the final volume up to 100 ml. The pH was then adjusted to 5.0. Stock solutions of auxins such as IAA, 2,4-D, NAA, and IBA were made by dissolving them in 70% ethanol, as these hormones are poorly soluble in water. On the other hand, stock solutions of cytokinins such as BA and KN were made by dissolving them in hydrochloric acid (HCl). Stock solutions using different plant growth hormones were prepared at a concentration of mg/l.

### Surface sterilization of explants

Following a 10-min incubation period in a Tween-20 solution (2 drops/100 ml), the explants (leaves, nodal, and internodal segments) were continuously stirred under tap water five to six times to eliminate the Tween-20. Then, the explants were treated with 0.1% mercuric chloride (HgCl_2_) by rinsing them in HgCl_2_ solution for 1–2 min, followed by 5–6 washes with autoclaved distilled water to remove any traces of HgCl_2_. The washing was carried out in the laminar air flow hood under sterilized conditions. Culture tubes containing MS medium, flasks, petri plates, forceps, distilled water (after autoclaving), and other materials like surgical blades (always used fresh), spirit lamp, scalpel, matchbox, cotton, parafilm, and gloves, except seeds and explants, were placed in the laminar airflow chamber for UV treatment for 30 min before inoculation. Before the experiment, the laminar air flow hood was sterilized using cleaned cotton dipped in 70% ethanol.

### Inoculation and incubation of explants

Leaves, nodal, and internodal segments (1–1.5 cm) were excised from 20-day-old plants and, after proper surface sterilization, placed in a Petri plate under laminar airflow. Transverse cuts on leaf margins and oblique cuts on nodal and internodal segments were made with the help of a pre-sterilized scalpel blade. These explants were then inoculated on a callus induction medium containing different concentrations of phytohormones in culture tubes. One explant per culture tube was inoculated. Twenty-four culture test tubes were set up for each experiment, and each set had three replicates. Before and after inoculation, the sides and rims of the culture tubes were flame sterilized. Culture tubes were sealed with aluminum foil and parafilm before placing them in culture tube stands under tissue culture laboratory conditions. The explants were incubated at room temperature (25 ± 2EC) and 75% relative humidity under white fluorescent lights for a 16 h/day photoperiod. The explants were monitored regularly to observe the induction of calli and to remove any contaminated cultures.

### Callus induction and multiplication

Aseptic callus cultures were established using two plant growth regulators (PGRs): auxin (2,4-dichlorophenoxyacetic acid, 2,4-D) at concentrations of 0.1, 0.25, 0.5, and 1 mg/l, and cytokinin (6-benzylaminopurine, BA) at concentrations of 0.25, 0.5, 0.75, and 1 mg/l. These were used both alone and in combinations (0.5 + + 0.5 mg/l; 0.5 + 0.75 mg/l; 0.75 + 0.5 mg/l; and 1 + + 1 mg/l of BA + 2,4-D, respectively) for callus induction and multiplication. Callus was induced in explants under different concentrations of these plant growth hormones.

### Shoot and root regeneration from callus

For root induction, IAA at concentrations of 0.5, 0.75, and 1 mg/l, IBA at concentrations of 0.25, 0.5, and 0.75 mg/l, and NAA at concentrations of 0.5, 0.75, and 1 mg/l were used alone. For shoot induction, 6-benzylaminopurine (BA) alone at concentrations of 0.25, 0.5, 0.75, and 1 mg/l and in combination with IAA at concentrations of 0.5 + 0.5 mg/l, 0.5 + + 0.75 mg/l, 0.75 + + 0.5 mg/l, and 1 + 1 mg/l of IAA + BA, respectively, were used. Additionally, Kinetin (KN) at concentrations of 0.1, 0.25, 0.5, and 1 mg/l was also used for shoot induction. Healthy callus induced from different explants were selected and cultured on an MS medium containing different concentrations of plant growth regulators (mg/l) for shoot regeneration. For root induction, regenerated shoots were first separated and then cultured on an MS medium containing different concentrations (0.50, 0.75, and 1.0 mg/l) of IAA, IBA, and NAA.

### Calculations of callus induction and regeneration

#### Callus induction percentage

Callus induction was carried out using different explants viz. intermodal segments, leaves, and nodal segments by inoculating them on MS medium supplemented with 6-Benzylaminopurine (BA), 2,4-dichlorophenoxyacetic acid (2,4-D), kinetin (KN), indole-3-acetic acid, indole-3-butyric acid, and 1-NAA. % callus induction was calculated as:


$$\text{Callus}\;\text{induction}[\%]=\frac{\text{Number of explants initiating callus}}{\text{Total number of explants}}\times 100$$ (1)

#### Callus multiplication

Induced callus was subcultured on a fresh medium consisting of varied concentrations of plant growth hormones for multiplication. After 30 days, the experiment was terminated, and the percentage of callus multiplication was calculated as follows:


$$\text{Callus}\;\text{multiplication}[\%]=\frac{\text{Number}\;\text{of}\;\text{explants}\;\text{initiating}\;\text{callus}\;\text{multiplication}}{\text{Total}\;\text{number}\;\text{of}\;\text{explants}}\times 100$$ (2)

#### Fresh weight of callus

The callus treated with different concentrations of PGRs was scraped off from the culture tube. It was then pressed gently on filter paper and transferred to a Petri plate. The average weight of three callus cultures grown in different test tubes was taken for the calculation of the fresh weight of the callus. Each experiment was set up in triplicates.


$$\text{Average}\;\text{fresh}\;\text{weight}[g]=\frac{\text{Total}\;\text{fresh}\;\text{weight}\;\text{of}\;\text{callus}\;\text{in}\;\text{the}\;\text{cultures}}{\text{Total}\;\text{number}\;\text{of}\;\text{cultures}}$$ (3)

#### Dry weight of the callus

Fresh callus measured in the Petri plate was kept in an oven for drying at 80#x00B0;C for 8–10 h. The dry weight of three callus cultures grown in each replicate was taken for the calculation and was measured as follows: Weight of empty Petri plate ! Weight of Petri plate containing dried callus


$$\text{Average}\;\text{dry}\;\text{weight}[g]=\frac{\text{Total}\;\text{dry}\;\text{weight}\;\text{of}\;\text{callus}\;\text{in}\;\text{the}\;\text{cultures}}{\text{Total}\;\text{number}\;\text{of}\;\text{cultures}}$$ (4)

#### Callus dry matter content

The callus dry matter content of three callus cultures grown in each replicate was calculated by the formula given by Khater et al. (2013) and was determined as follows:


$$\text{Dry}\;\text{matter}\;\text{content}[\%]=\frac{\text{Dry}\;\text{weight}\;\text{of}\;\text{callus}}{\text{Fresh}\;\text{weight}\;\text{of}\;\text{callus}}\times 100$$ (5)

#### Shoot regeneration

The shoot regeneration percentage was calculated as follows:


$$\text{Shoot}\;\text{regeneration}[\%]=\frac{\text{Number}\;\text{of}\;\text{cultures}\;\text{with}\;\text{shoots}\;\text{regenerated}}{\text{Total}\;\text{number}\;\text{of}\;\text{cultures}}\times 100$$ (6)

#### Shoot number

After 30 days, shoots were taken out from the MS medium and cleaned gently with the help of filter paper. The shoot number was counted individually for three test tubes, and the mean was calculated.

#### Shoot length

Shoot lengths of three callus cultures grown in each replicate were measured in centimeters using a meter rule from each replication and results were presented as mean ± S.E.

#### Root regeneration

The root regeneration percentage was calculated as follows:


$$\text{Root}\;\text{regeneration}[\%]=\frac{\text{Number}\;\text{of}\;\text{cultures}\;\text{with}\;\text{roots}\;\text{regenerated}}{\text{Total}\;\text{number}\;\text{of}\;\text{cultures}}\times 100$$ (7)

#### Root number

After 30 days, plants were taken out from the MS medium and cleaned gently with the help of filter paper. The root number was counted individually for three test tubes, and the mean was calculated.

#### Root length

The average root lengths of three roots grown in each replicate were measured in centimeters using a meter rule, and results were presented as mean ± S.E.

### Statistical analysis

The data was represented in terms of mean with standard error and results were analyzed by employing Analysis of Variance (One Way ANOVA) and Tukey’s test (SPSS version 16.0, SPSS Inc., Chicago, IL).

## Results

### Effects on callus growth parameters

#### Callus induction [%]

Maximum callus induction (77.77%) from leaves was observed in the MS medium with 0.75 mg/l of BA. In contrast, the lowest callus induction (62.50%) was achieved with 0.25 mg/l of BA. When leaves were employed as explants, MS media with 0.5 mg/l of 2,4-D demonstrated a maximum callus induction of 75%. The MS medium containing 0.50 + 0.75 mg/l of BA + 2,4-D showed the highest callus induction of 79.16%, followed by 69.44% at 0.50 + 0.50 mg/l of BA + 2,4-D.

For nodal segments, maximum callus induction (79.16%) was seen in the MS medium containing 0.75 mg/l of BA. The lowest callus induction (61.11%) was achieved with 0.25 mg/l of BA. The maximum callus induction (83.33%) was obtained using the MS medium with 0.25 mg/l of 2,4-D. The MS medium containing 0.50 + 0.50 mg/l of BA + 2,4-D produced an average callus induction of 81.94%, while the MS medium containing 1.0 + 1.0 mg/l of BA + 2,4-D produced a callus induction of 73.61%. A one-way ANOVA demonstrating callus induction under BA, 2,4-D, and BA + 2,4-D treatments revealed significant differences at a significance level of *P* < 0.05.

**Fig. 1:**
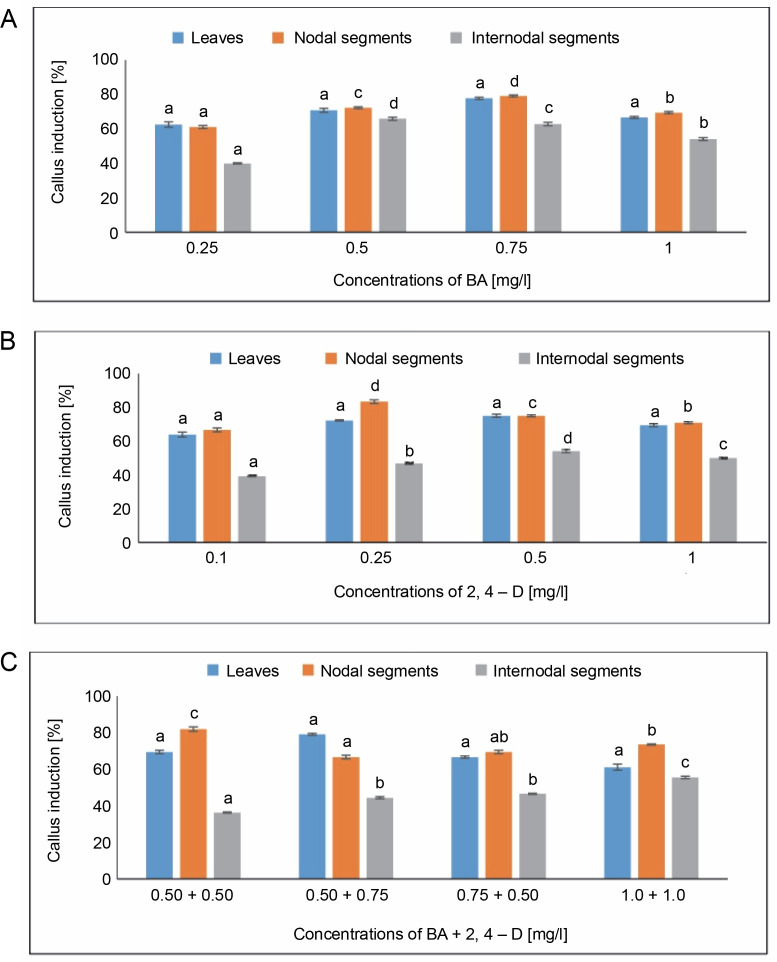
Effects of (A) 6-benzylaminopurine (BA), (B) 2,4-dichlorophenoxyacetic acid (2,4-D) and (C) BA + 2,4-D on callus induction percentage callus induced from leaves, nodal and internodal segments of *Solanum lycopersicum* after 30 days; abc – statistically significant differences (One-way ANOVA, Tukey’s test, *P* ≤ 0.05) are indicated by the different letters with the above mean values of percent callus induction from leaves, nodal, and internodal segments

For internodal segments, the maximum callus induction (65.90%) was found in the MS medium containing 0.50 mg/l of BA, whereas the minimum callus induction (40%) was found in the MS medium containing 0.25 mg/l of BA. Callus induction (54.05%) was obtained with 0.5 mg/l of 2,4-D. In the MS medium containing 1.0 + + 1.0 mg/l of BA + 2,4-D, a callus induction rate of 55.5% was achieved, followed by 46.66% in the MS medium containing 0.75 + 0.50 mg/l of BA + 2,4-D (Fig. 1).

#### Callus multiplication [%]

The highest percentage of callus multiplication, 83.33%, was seen in MS medium with 0.75 mg/l of BA. However, when leaves were employed as explants, the lowest percentage of callus multiplication (65.21%) was achieved in MS media containing 0.25 mg/l of BA. The percentage of callus multiplication in MS media containing 0.5 mg/l of 2,4-D was reported as 59%. An average of 82.45% callus multiplication was obtained at 0.50 + 0.75 mg/l in MS medium containing BA + 2,4-D, followed by 72% in MS medium containing 0.50 + + 0.50 mg/l from leaves.

The MS medium containing 0.75 mg/l showed the largest percentage of callus multiplication (82.45%) from nodal segments. In comparison, a minimum percentage of callus multiplication (65.90%) was achieved in MS media containing 0.25 mg/l of BA. An analysis using MS media containing 0.25 mg/l of 2,4-D showed an 86.66% callus multiplication. For BA + 2,4-D in the MS medium, the highest percentage of callus multiplication (61.11%) was seen in the MS medium containing 0.50 mg/l of BA.

**Fig. 2:**
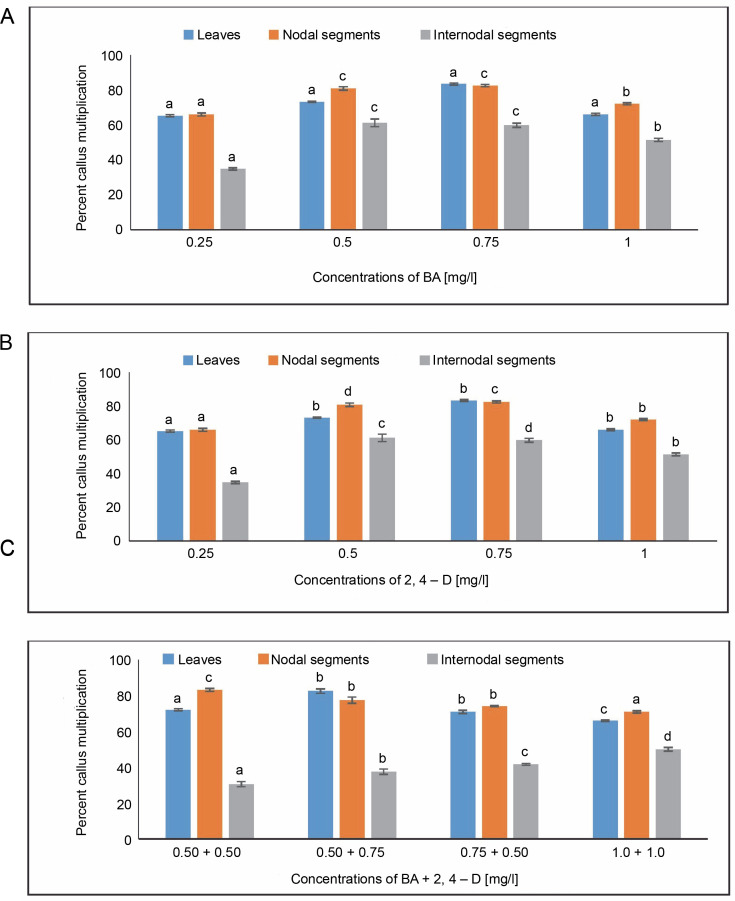
Effects of (A) 6-benzylaminopurine (BA), (B) 2,4-dichlorophenoxyacetic acid (2,4-D), and (C) BA + 2,4-D on callus multiplication of callus induced from leaves, nodal and internodal segments of *Solanum lycopersicum* after 30 days; abc – statistically significant differences (One-way ANOVA, Tukey’s test, *P* ≤ 0.05) are indicated by the different letters with the above mean values of percent callus induction from leaves, nodal, and internodal segments

From internodal segments, the highest percentage of callus multiplication (83.05%) was obtained at 0.50 + + 0.50 mg/l, followed by 77.35% in MS medium containing 0.50 + 0.75 mg/l. A 51.38% callus multiplication percentage was reported with 0.5 mg/l of 2,4-D in MS medium. A callus multiplication percentage of 50% was attained at 1.0 + 1.0 mg/l in the case of BA + 2,4-D in MS-supplemented medium, and 41.66% at 0.75% + + 0.50 mg/l (Fig. 2). Using One-way ANOVA and Tukey’s test, variations in the percentage of callus multiplication for the specified treatments under BA, 2,4-D, and BA + + 2,4-D treatment were shown to be statistically significant.

#### Fresh weight of callus

**Fig. 3:**
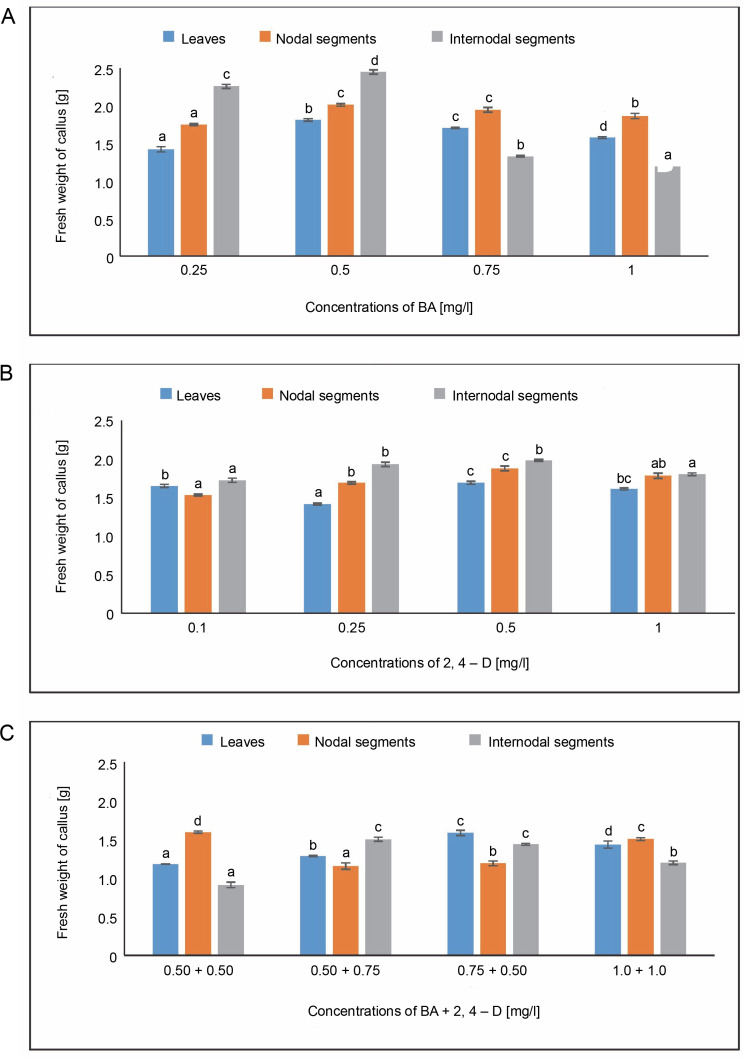
Effects of (A) 6-benzylaminopurine (BA), (B) 2,4-dichlorophenoxyacetic acid (2,4-D), and (C) BA + 2,4-D on fresh weight of callus induced from leaves, nodal and internodal segments of *Solanum lycopersicum* after 30 days; abcd – statistically significant differences (One-way ANOVA; Tukey’s test, *P* ≤ 0.05) are indicated by the different letters with the above mean values of percent callus induction from leaves, nodal, and internodal segments

The MS medium containing 0.50 mg/l of BA yielded the largest fresh weight of callus (1.811 g) from leaves, while the MS medium containing 0.25 mg/l of BA produced the lowest fresh weight of callus (1.421 g). In MS media containing 0.5 mg/l of 2,4-D, 1.693 g of fresh callus was measured. The maximal fresh weight of callus in the BA + 2,4-D case was 1.581 g in MS medium containing 0.75 + 0.50 mg/l and 1.432 g at 1.0 + 1.0 mg/l. In MS media containing 0.50 mg/l of BA, the fresh weight of callus produced from nodal segments was observed to be 2.010 g. The fresh weight of callus in the BA + 2,4-D case was 1.592 g in MS medium containing 0.50 + + 0.50 mg/l and 1.501 g at 1.0 + 1.0 mg/l. The maximum fresh weight of callus (2.448 g) from internodal segments was found in MS media containing 0.50 mg/l, whereas the minimal fresh weight of callus (1.193 g) was found at 1.0 mg/l of BA. Fresh callus weight, or 1.50 g, was attained in MS medium containing 0.50 + 0.75 mg/l in the BA + 2,4-D instance, followed by 1.435 g in MS medium containing 0.75 + 0.50 mg/l (Fig. 3). Using Oneway ANOVA, differences in the fresh weights of callus were shown to be statistically significant.

### Dry weight of callus

The lowest dry weight of callus, 0.094 g, was obtained in MS medium containing 0.25 and 1.0 mg/l of BA, while the largest dry weight of callus, 0.128 g, was detected from leaves in MS medium containing 0.75 mg/l. The callus’s maximal dry weight of 0.124 g was attained with 2,4-D treatment. The maximum dry weight of callus in the BA + 2,4-D case was 0.111 g in MS medium containing 0.50 + 0.75 mg/l, followed by 0.093 g at 0.75 + + 0.50 mg/l.

**Fig. 4:**
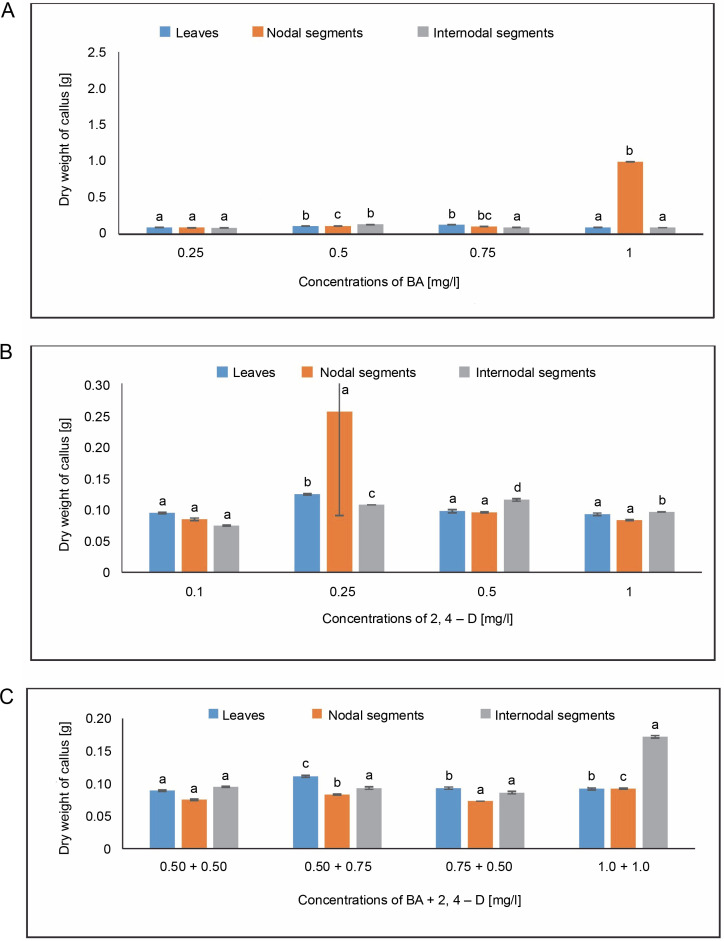
Effects of (A) 6-benzylaminopurine, (B) 2,4-dichlorophenoxyacetic acid (2,4-D), and (C) BA + 2,4-D on dry weight of callus induced from leaves, nodal, and internodal segments of *Solanum lycopersicum* after 30 days; abc – statistically significant differences (One-way ANOVA, Tukey’s test, *P* ≤ 0.05) are indicated by the different letters with the above mean values of percent callus induction from leaves, nodal, and internodal segments

In the instance of BA + 2,4-D, a dry weight of 0.092 g was achieved in MS medium containing 1.0 + + 1.0 mg/l, followed by 0.083 g in MS medium containing 0.50 + 0.75 mg/l. The MS medium containing 0.50 mg/l showed the largest dry weight of the callus, 0.132 g, from internodal segments, while the MS medium containing 0.25 and 1.0 mg/l of BA produced the lowest dry weight of the callus, 0.084 g. The maximum dry weight of the callus under BA + 2,4-D treatment was 0.172 g in MS medium containing 1.0 + 1.0 mg/l, and the second-highest dry weight was 0.095 g at 0.50 + + 0.50 mg/l (Fig. 4).

#### Callus dry matter content

In MS media containing 0.75 mg/l of BA, the maximum callus dry matter content produced from leaves was observed to be 7.536%. In contrast, the MS medium containing 1.0 mg/l of BA produced the least callus dry matter content (6.005%). An 8.775% callus dry matter concentration was found in MS media containing 0.25 mg/l of 2,4-D. For BA + 2,4-D, the callus dry matter content was 8.710% in MS medium with 0.50 + + 0.75 mg/l and 7.595% at 0.50 + 0.50 mg/l.

In MS medium containing 1.0 mg/l of BA, callus dry matter content of 53.43% was detected from nodal segments. In MS media with 0.25 mg/l of 2,4-D, a 15.04% dry matter amount was found in the callus. In MS media containing 0.50 + 0.75 mg/l, a callus dry matter concentration of 7.249% was recorded with BA + 2,4-D treatment.

The MS medium containing 1.0 mg/l of BA showed the greatest callus dry matter concentration (7.478%) from internodal segments. The dry matter content of the callus was found to be 5.804% in an MS medium containing 0.50 mg/l of 2,4-D. The maximum callus dry matter content (14.25%) was achieved after BA + 2,4-D treatment in MS medium containing 1.0 + 1.0 mg/l.

**Fig. 5:**
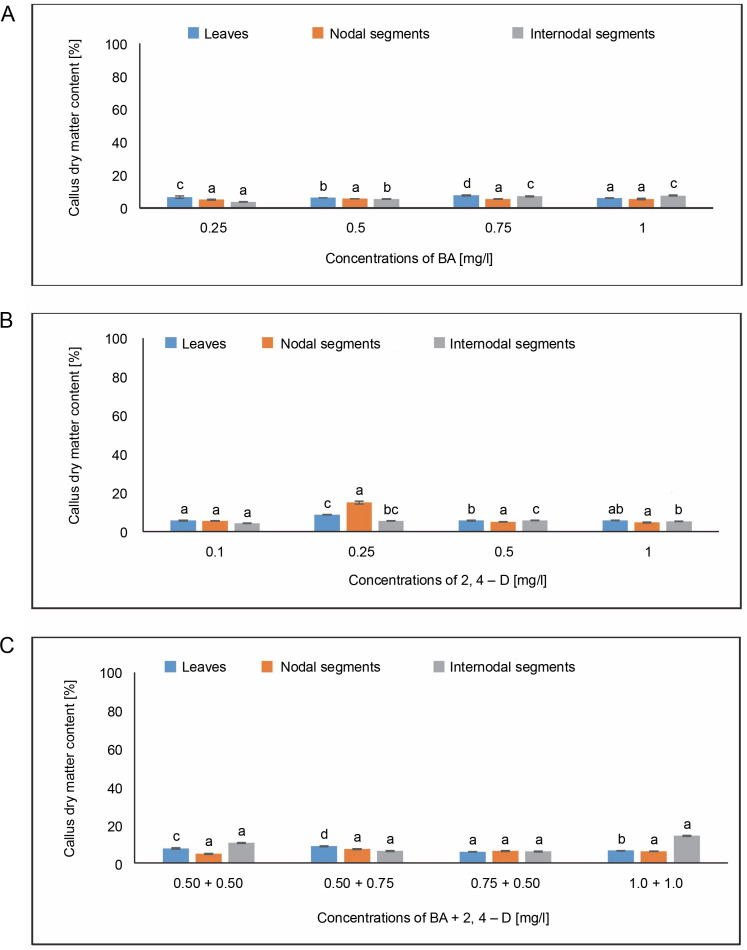
Effects of (A) 6-benzylaminopurine (BA), (B) 2,4-dichlorophenoxyacetic acid (2,4-D), and (C) BA + 2,4-D on callus dry matter content induced from leaves, nodal, and internodal segments of *Solanum lycopersicum* after 30 days; abc – statistically significant differences (One-way ANOVA, Tukey’s test, *P* ≤ 0.05) are indicated by the different letters with the above mean values of percent callus induction from leaves, nodal, and internodal segments

Statistical analysis showed significant differences among callus dry matter content at different concentrations of BA, 2,4-D, and BA + 2,4-D, which were statistically significant at *P* ≤ 0.05 (level of significance) (Fig. 5).

### Effects on callus regeneration dynamics

#### Shoot induction [%]

**Fig. 6:**
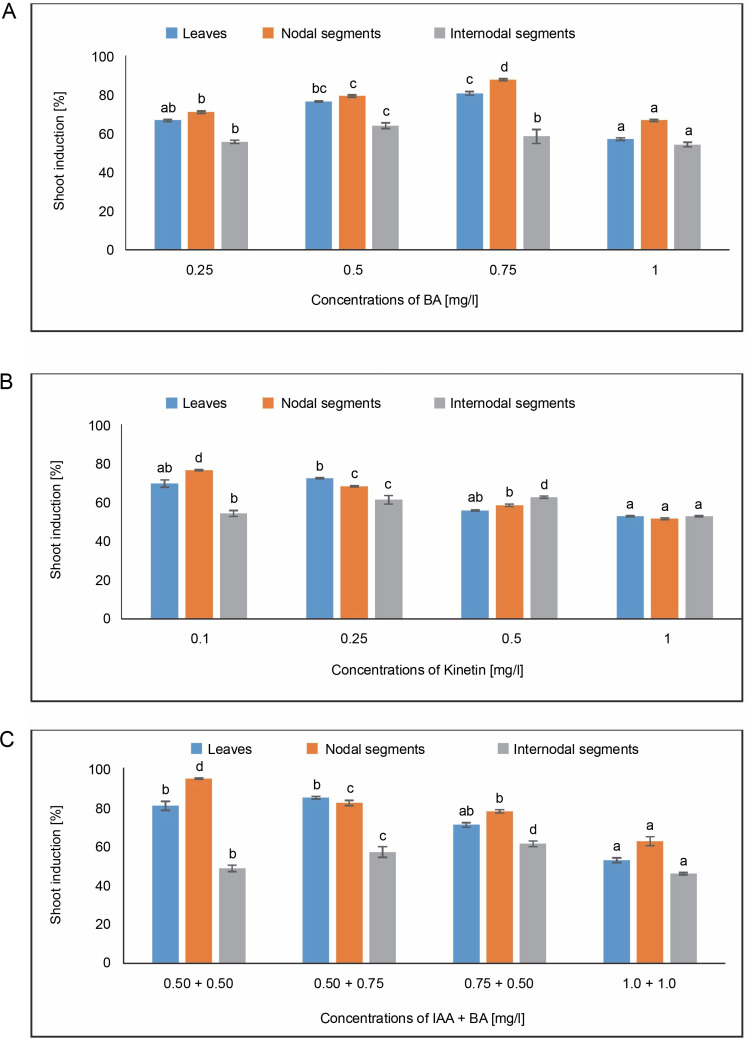
Effects of (A) 6-benzylaminopurine (BA), (B) Kinetin, and (C) Indole acetic acid (IAA + BA) on shoot induction (%) of callus induced from leaves, nodal, and internodal segments of *Solanum lycopersicum* after 30 days; abc – statistically significant differences (One-way ANOVA, Tukey’s test, *P* ≤ 0.05) are indicated by the different letters with the above mean values of percent callus induction from leaves, nodal, and internodal segments

Figure 6 displays the results of shoot induction (%). The highest shoot induction, 80.55%, was observed in callus produced from leaves in MS medium containing 0.75 mg/l of BA. A 72.22% shoot induction was obtained in MS medium containing 0.25 mg/l of KN. The MS medium comprising 0.50 + 0.75 mg/l produced the maximum shoot induction (84.72%) in the IAA + BA condition, while the MS medium having 0.50 + 0.50 mg/l produced 80.52% shoot induction. In MS medium containing 0.75 mg/l of BA, 87.50% shoot induction was seen.

In callus induced from nodal segments, 66.66% shoot induction was noted in MS media containing 1.0 mg/l of BA. A 76.38% shoot induction was achieved with 0.1 mg/l of KN treatment. For IAA + BA, shoot induction was achieved at 0.50 + 0.50 mg/l, yielding 94.44%, and at 0.50 + 0.75 mg/l, yielding 81.94%. Maximum shoot induction (63.88%) was observed in callus produced from internodal segments in MS medium containing 0.50 mg/l of BA. Six 2.50% shoot induction was noted in MS medium containing 0.50 mg/l of KN. The MS medium containing 0.75 + 0.50 mg/l showed the maximum shoot induction (61.11%) in the IAA + BA condition.

In the current investigation, significant differences were observed at the *P* < 0.05 level of significance in the one-way ANOVA for shoot induction (%) under the BA, Kinetin, and IAA + BA treatments.

#### Shoot number

In MS media containing 0.75 mg/l of BA, callus produced from leaves revealed a shoot number of 2.444. When treated with 0.25 mg/l of KN, the maximum shoot number of 1.66 was noted. The average shoot number (3.33) for IAA + BA was found in the MS medium containing 0.50 + 0.50 mg/l, while 1.66 was found in the MS medium having 0.75 + 0.50 mg/l. A shoot number of 3.111 was seen during BA treatment at a dosage of 0.75 mg/l.

**Fig. 7:**
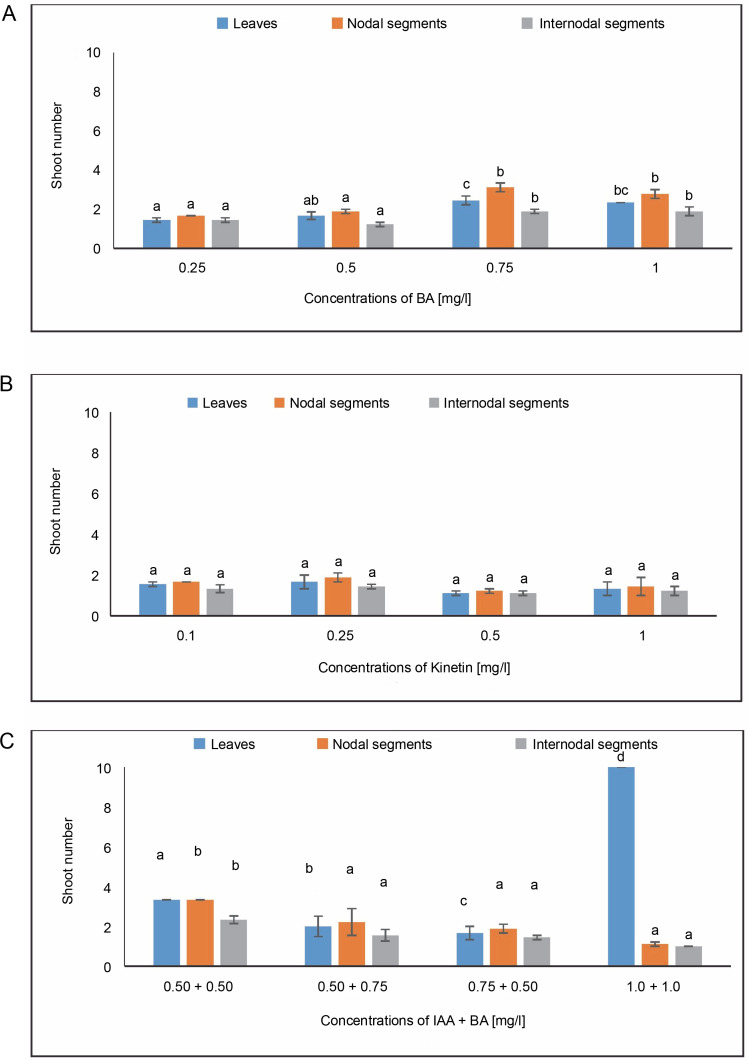
Effects of (A) 6-benzylaminopurine (BA), (B) Kinetin, and (C) Indole acetic acid (IAA + BA) on shoot number of callus induced from leaves, nodal, and internodal segments of *Solanum lycopersicum* after 30 days; abc – statistically significant differences (One-way ANOVA, Tukey’s test, *P* ≤ 0.05) are indicated by the different letters with the above mean values of percent callus induction from leaves, nodal, and internodal segments

Nodal segments yielded the largest shoot number (1.88) in MS media containing 0.25 mg/l of KN. The maximal shoot number (3.333) for IAA + BA was achieved in MS medium containing 0.50 + 0.50 mg/l, and 2.222 at 0.50 + 0.75 mg/l. Internodal segments-induced callus revealed a shoot number of 1.888 in MS media with 0.75 mg/l of BA. In MS media containing 0.25 mg/l during KN treatment, an average shoot number of 1.444 was achieved. The greatest shoot number for IAA + BA was 2.333 in MS medium having 0.50 + 0.50 mg/l, and the next highest shoot number was 1.555 in MS medium containing 0.50 + 0.75 mg/l (Fig. 7). One way ANOVA for shoot number under BA and IAA + BA treatments during the current investigation revealed significant changes at *P* ≤ 0.05. (level of significance).

#### Shoot length

**Fig. 8:**
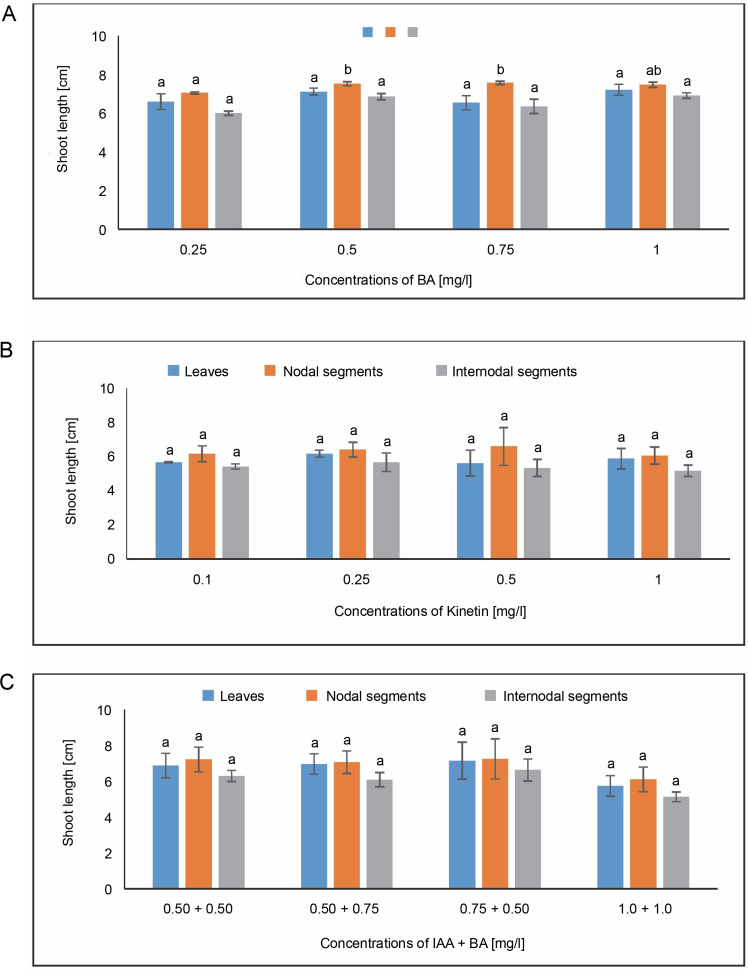
Effects of (A) 6-benzylaminopurine (BA), (B) Kinetin, and (C) Indole acetic acid (IAA + BA) on shoot length of callus induced from leaves, nodal, and internodal segments of *Solanum lycopersicum* after 30 days; abc – statistically significant differences (One-way ANOVA, Tukey’s test, *P* ≤ 0.05) are indicated by the different letters with the above mean values of percent callus induction from leaves, nodal, and internodal segments

In MS medium containing 1.0 mg/l, callus produced from leaves demonstrated an average shoot length of 7.222 cm, while the minimum shoot length of 6.544 cm was obtained in MS medium containing 0.75 mg/l of BA. The average shoot length of 6.177 cm was found at a concentration of 0.25 mg/l of KN. The maximum shoot length of 7.144 cm was seen in the IAA + BA instance in MS media having 0.75 + 0.50 mg/l, followed by 6.956 cm in MS mixture containing 0.50 + 0.75 mg/l. The maximum shoot length of 7.570 cm from nodal segments was found in the MS medium with 0.75 mg/l of BA. An average shoot length of 6.604 cm was observed at a KN concentration of 0.50 mg/l. The average shoot length (7.245 cm) for IAA + BA was achieved in MS medium with 0.75 + 0.50 mg/l. When callus was generated from internodal segments, the average shoot length in MS media containing 1.0 mg/l was 6.912 cm, while the average shoot length in MS medium containing 0.25 mg/l of BA was 6.002 cm. The highest shoot length of 6.622 cm was achieved in the IAA + BA instance in MS media containing 0.75 + 0.50 mg/l, and 5.124 cm at 1.0 + 1.0 mg/l (Fig. 8). Statistical analysis revealed a substantial variation in the length of the shoots.

#### Root induction [%]

**Fig. 9:**
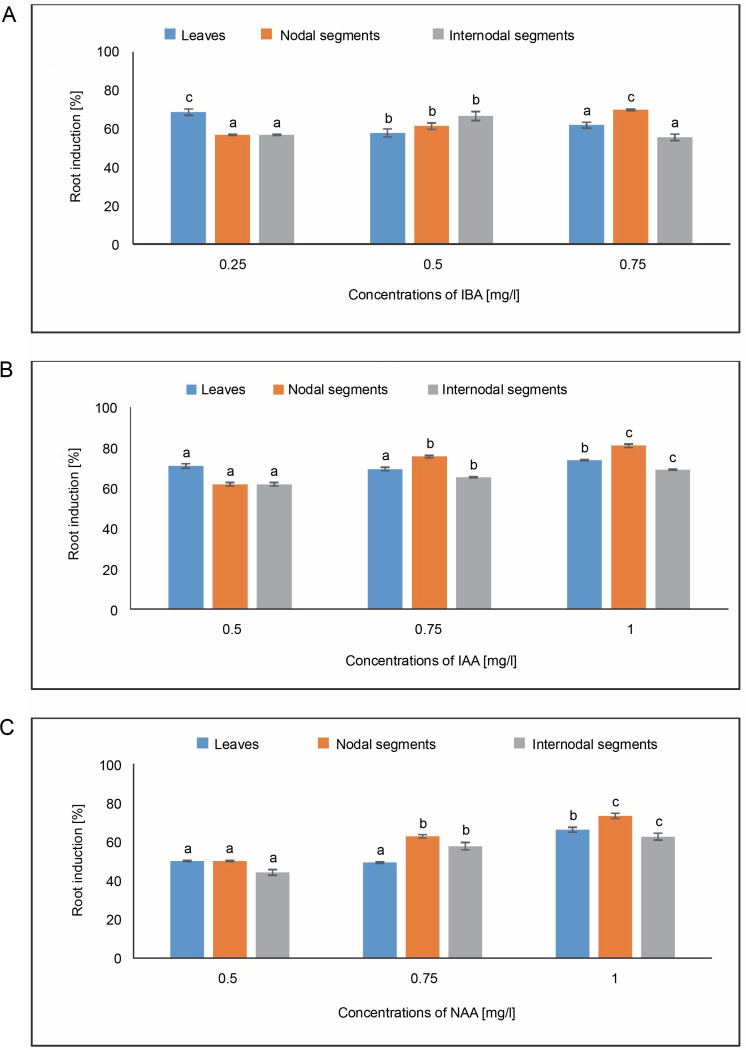
Effects of (A) Indole butyric acid (IBA), (B) Indole acetic acid (IAA), and (C) Naphthalene acetic acid (NAA) on root induction (%) of callus induced from leaves, nodal, and internodal segments of *Solanum lycopersicum* after 30 days; abc – statistically significant differences (One-way ANOVA, Tukey’s test, *P* ≤ 0.05) are indicated by the different letters with the above mean values of percent callus induction from leaves, nodal, and internodal segments

As shown in Figure 9, callus produced from leaves showed root induction of 68.62% in MS media containing 0.25 mg/l of IBA. The maximum root induction of 73.80% was seen in the MS medium containing 1.0 mg/l of IAA. In the case of NAA, 50% root induction at 0.50 mg/l and an average root induction of 66.07% were obtained in an MS medium containing 1.0 mg/l. The MS medium containing 0.75 mg/l of IBA from callus produced from nodal segments showed the highest root induction of 69.85%. An average root induction of 80.95% was achieved at 1.0 mg/l of IAA in the MS medium. Maximum root induction of 73.21% was achieved in the case of NAA in MS media containing 1.0 mg/l, and 62.71% at 0.75 mg/l. Root induction of 66.66% was observed in callus produced from internodal segments in MS media containing 0.50 mg/l of IBA. It was possible to produce 80.95% root induction using an MS medium containing 1.0 mg/l of IAA. One-way ANOVA and Tukey’s test revealed that differences in root induction (%) for the specified treatments under IBA, IAA, and NAA were statistically significant.

#### Root number

**Fig. 10:**
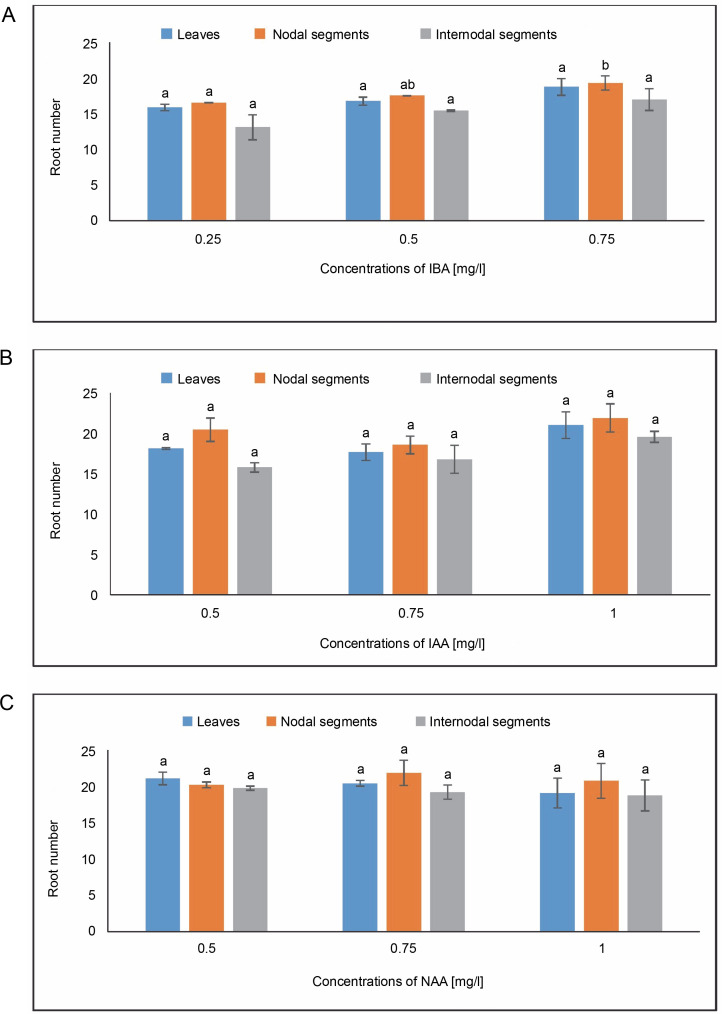
Effects of (A) Indole butyric acid (IBA), (B) Indole acetic acid (IAA), and (C) Naphthalene acetic acid (NAA) on root number of callus induced from leaves, nodal, and internodal segments of *Solanum lycopersicum* after 30 days; abc – statistically significant differences (One-way ANOVA, Tukey’s test, *P* ≤ 0.05) are indicated by the different letters with the above mean values of percent callus induction from leaves, nodal, and internodal segments

In MS media with 0.75 mg/l of IBA, callus produced from leaves showed an average root number of 18.77. IAA at a concentration of 0.75 mg/l in MS medium produced an average root number of 18.55. The largest root number, 20.44, was achieved at 0.75 mg/l in an MS medium containing NAA, followed by 19.11 at 1.0 mg/l. The average root number in the MS medium containing 0.75 mg/l of IBA was 19.33, and the average root number of 21.88 was found in the MS medium containing 1.0 mg/l of IAA. In the instance of NAA, the greatest root number, 21.88, was obtained in MS media containing 0.75 mg/l, while the callus produced from nodal segments yielded 20.77 at 1.0 mg/l. In MS media containing 0.75 mg/l, the callus produced from internodal segments had an average root number of 17, whereas the minimum root number was 13.11 at 0.25 mg/l of IBA. The greatest root number, 19.77, was found in the case of NAA at 0.50 mg/l, and 19.22 in the MS medium containing 0.75 mg/l (Fig. 10).

#### Root length

**Fig. 11:**
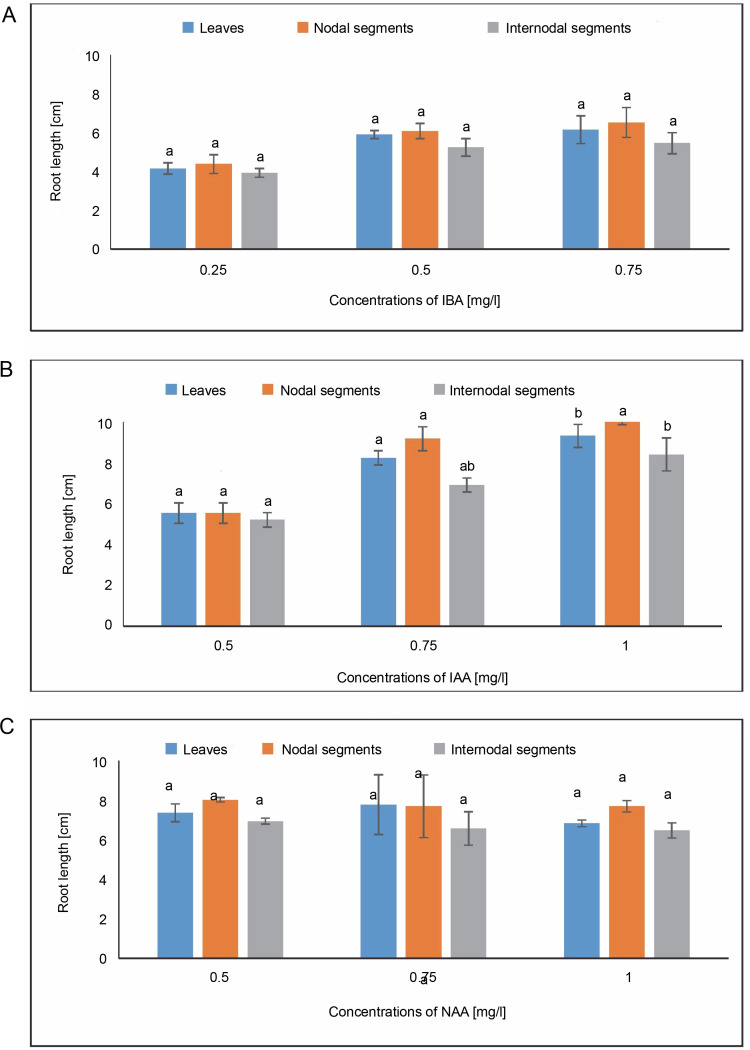
Effects of (A) Indole butyric acid (IBA), (B) Indole acetic acid (IAA), and (C) Naphthalene acetic acid (NAA) on root length of callus induced from leaves, nodal, and internodal segments of *Solanum lycopersicum* after 30 days; abc – statistically significant differences (One-way ANOVA, Tukey’s test, *P* ≤ 0.05) are indicated by the different letters with the above mean values of percent callus induction from leaves, nodal, and internodal segments

The highest root length of 6.143 cm was observed in roots produced in the callus in MS medium containing 0.75 mg/l of IBA. The average root length measured in MS medium at a concentration of 1.0 mg/l of IAA was 9.312 cm. The maximum root length under NAA treatment was 7.766 cm in MS media containing 0.75 mg/l, while the second-highest root length was 7.355 cm at 0.50 mg/l. The maximal root length, or 6.515 cm, was demonstrated by a callus produced from nodal segments in MS media containing 0.75 mg/l of IBA. The average root length (8.012 cm) in the NAA treatment condition was measured in MS media containing 0.50 mg/l. An average root length of 8.401 cm was achieved at a concentration of 1.0 mg/l of IAA. When it came to NAA, the longest root length (6.933 cm) was seen in the MS medium with 0.50 mg/l, and the next-longest root length (6.566 cm) was seen at 0.75 mg/l. Using One-way ANOVA and Tukey’s test, the variations in root induction (%) variations were shown to be statistically significant for the specified treatments under IAA (Fig. 11).

## Discussion

During the present study, the standardization of a protocol for micropropagation of *S. lycopersicum* and the establishment of aseptic cultures from different explants (leaves, nodal, and internodal segments) of *S. lycopersicum* were achieved.

### Callus induction and regeneration through leaves

Atulkar et al. (2012) conducted a study on *Coleus forskohlii* using leaf explants for callus induction and shoot regeneration. Their study revealed that a combination of BA and 2,4-D (0.5 + 1.0 mg/l) was the best for callus induction and proliferation. Our study yielded similar results, with maximum callus induction observed when BA and 2,4-D were used in combination (0.5 + + 0.75 mg/l). Similar findings were reported for *Holarrhena antidysenterica* by Raha et al. (2003).

Colgecen et al. (2012) reported that the application of cytokinins resulted in the most effective callus induction for *Centaurea tchihatcheffii* Fisch. Our results are also in agreement with Jatoi et al. (2001), who reported maximum green callus of tomato on callus induction media containing 1 mg/l BAP and 0.1 mg/l IAA. Jan et al. (2015) studied the effect of PGRs on callus induction and regeneration in tomatoes and observed a maximum callus induction frequency of 90% on MS media containing 4 mg/l 2,4-D and 0.5 mg/l BAP.

Different hormones like BAP and IAA, and IAA and KN were used in combinations for callus induction and regeneration (Chen et al., 1999). The maximum callus formation (91.2%) was observed when leaves were used as explants in *Tecomella undulata* on MS medium supplemented with BAP and 2,4-D at (3.0 + 0.5 mg/l) and (90.4%) at (2.5 + 0.5 mg/l) or 2,4-D alone at 3.0 mg/l (Patel and Patel, 2013). Similarly, Hassan et al. (2019) reported that callus derived from leaf explant on MS medium containing 0.25 mg/l 2,4-D gained the highest fresh weight for *Ocimum basilicum*. Callus induction and regeneration in *Lilium leucanthum* were studied by Tang et al. (2010), who observed that MS medium with 1.0 mg/l BA and 1.0 mg/l 2,4-D was optimal for callus induction from leaf explants. Similar to our results, Gopi and Vatsala (2006) reported callus induction in *Gymnema sylvestre* under MS medium containing 0.5 mg/l 2,4-D.

During the present study, both the fresh weight and dry weight of calli varied significantly due to different concentrations and combinations of hormones. Leaf explants cultured on the MS medium containing 0.5 mg/l of BA produced the maximum fresh weight of callus, while the MS medium containing 3 mg/l of BAP + + 0.25 mg/l of NAA produced the maximum dry weight of callus. Similar observations were reported by Papry et al. (2015) in the case of induction of callus from stem explants of tomato, where the maximum fresh and dry weight of callus after 30 days was observed under a combination of 3 mg/l BAP + 0.25 mg/l NAA. Capote et al. (2000) made similar observations with stem segments and leaf tissue of five cultivars of tomato when grown in a medium containing BAP and NAA. Maximum callus biomass induced from leaves (2.36 g fresh weight and 1.238 g dry weight) in MS media containing 3.0 mg/l of BAP + 0.5 mg/l of 2,4-D was observed by Patel and Patel (2013). During the present study, maximum callus dry matter content was observed in MS medium containing 0.75 mg/l, 0.25 mg/l of 2,4-D, and 0.50 mg/l of BA + + 0.75 mg/l of 2,4-D.

The present work indicated maximum shoot induction in MS medium containing 0.75 mg/l BA, 0.25 mg/l Kinetin, and 0.50 mg/l of IAA + 0.75 mg/l of BA. Girish and Katiyar (2000) evaluated various combinations of phytohormones and reported that MS medium with 1.5 mg BAP and 1.5 mg IAA/l was the most responsive medium for regeneration in *Lycopersicon esculentum*. The presence of two cytokinins (BAP at 5.0 mg/l and Zeatin at 1.0 mg/l) showed the best results in terms of *L. esculentum* plant regeneration, as reported by Brichkova et al. (2003). Shoot regeneration from the callus depended on various factors like composition of media, type of explant, genotype, quantity of media, light intensity and quality, plant growth hormones, media gelling agent, duration of light exposure, and temperature (Bhatia et al. 2004).

The present study revealed the maximum shoot number at 0.50 + 0.5 mg/l of IAA + BA. Maximum shoot length was observed in MS medium containing 0.75 mg/l of BA when nodal segments were used as explants. The type of explants determined the incidence of the explant’s organogenesis and shoot number per leaf explant (Bahurpe et al., 2013). The maximum number of shoots in tomato was obtained as 3 and 3.5, produced when leaves were used as explants at 2.0 mg/l BAP, and the number of shoots progressively increased with the increased duration of hormonal treatments (Papry et al., 2016).

The present study indicated maximum root induction at 0.25 mg/l, at 1.0 mg/l IAA, and in MS medium containing 1.0 mg/l of NAA. Contrary to this, Rashid and Bal (2010) reported that tomato plants did not require any exogenous plant growth regulators for root induction as they observed 100% rooting of shoots without any plant growth regulators in MS medium. However, certain hormones like auxins (IAA, IBA, and NAA), alone or in combinations, were used for rooting tomato shoots at varying concentrations from 0.1 to 1.0 mg/l by Sherkar and Chavan (2014). They reported that the addition of IBA (2.0 mg/l) was found essential to induce profuse roots in tomatoes. The maximum number of shoots and fresh weight of tomato was observed in a medium containing 8 mg/l BA and 4 mg/l BA, respectively, with decreased shoot and internode length with increasing BA concentration.

The present study revealed the maximum root number at 0.75 mg/l, in MS medium containing 1.0 mg/l IAA and MS medium containing 0.75 mg/l of NAA when nodal segments were used as explants. The present work revealed a minimum number of roots at 0.25 mg/l of IBA. Similarly, the lowest number of roots was observed on a half-strength MS medium with 0.1 mg/l IAA in *S. lycopersicum* (Papry et al., 2016).

### Callus induction and regeneration through nodal segments

During the present work, maximum shoot induction was observed at 0.50 + 0.50 mg/l IAA + BA. Savita et al. (2010) reported that the best callus regeneration response in *Citrus jambhiri* was 71% with nodal segments in MS medium supplemented with NAA (0.5 mg/l) and BA (3 mg/l). BA and NAA were also reported to be favorable for shoot regeneration from calli of different citrus species (Beloualy, 1991), whereas the use of BA alone was proven as an efficient treatment for shoot regeneration in different *Citrus* spp. (Costa et al., 2002). Maximum shooting response from nodal segments in *Coleus blumei* was observed in 0.2 mg/l IBA (Rani et al. 2006). Iqbal et al. (2019) reported that BA and NAA (in combination) at 4 and 5 mg/l concentrations, respectively, stimulated maximum callus production from nodal explants in potatoes.

In the present work, the maximum shoot number was observed at 0.50 + 0.50 mg/l of IAA + BA, and the maximum shoot length was observed at 0.75 mg/l of BA. Abu-Romman et al. (2015) reported Kinetin as a better plant growth regulator for shoot regeneration in cucumber plants. The highest shoot and internode length and the maximum number of shoots in *Lantana camara* were observed in a medium supplemented with 8 mg/l BA, and the highest fresh weight was obtained in 4 mg/l BA (Samani et al., 2014). Shoot production (1 shoot/explant) was observed through the growth of preexisting meristems from nodal segments of *Passiflora suberosa* cultured on MS medium amended with 2,4-D, PIC, and NAA (Garcia et al. 2011).

The present study indicated maximum root induction at 0.75 mg/l of IAA when tomato nodal segments were used as explants. Between NAA and IBA, which were used for rooting, NAA showed a better response (71%) compared to IBA. This aligns with the findings of Rani et al. (2006), who noted maximum rooting response from nodal segments in *Coleus blumei* in MS medium containing 2 mg/l of IBA.

In our study, the maximum root number and root length were observed in MS medium containing 0.75 mg/l of NAA and 1.0 mg/l of IAA, respectively. Similarly, Samani et al. (2014) reported the maximum number of roots in *L. camara* in a medium containing 0.5 mg/l of IAA. Hajong et al. (2013) found that the rooting of regenerated shoots of *Dendrobium chrysanthemum* was 100% with an average of 11.26 roots per shoot, while the mean root length (2.45 cm) was obtained in an MS medium containing 10 μM NAA.

Bernabe et al. (2012) reported that shoots could grow (96.9%) with 8.28 μM of IBA, having maximum root and shoot biomass in *Dioscorea remotiflora*.

### Callus induction and regeneration through internodal segments

According to the current investigation, the MS medium containing 0.50 mg/l of BA, 0.5 mg/l of 2,4-D, and 1.0 mg/l of BA + 1.0 mg/l of 2,4-D showed the highest callus induction. In MS media containing 0.50 mg/l of BA, 0.5 mg/l of 2,4-D, and 0.75 mg/l of BA + 0.50 mg/l of 2,4-D, the highest percentage of callus multiplication was seen. Abu-Romman et al. (2013) in their study on cucumber observed that the frequency of callus induction, its growth rate, and nature varied significantly depending on the type and concentration of the PGR used, and all the studied parameters (regeneration percentage, shoot length, shoot number) were higher for auxins compared to cytokinins.

The internodal segment of Grewia tenax (as explant) cultured on MS medium in combination with indole-3-butyric acid (IBA), 2,4-dichlorophenoxyacetic acid, or NAA was used for callus induction, and during the study, a yellow and friable callus was produced using 2.0 mg/l of NAA (Daffalla et al., 2019).

During the present study, the maximum fresh and dry weight of calli was observed at 0.50 mg/l of BA and 1.0 + 1.0 mg/l of BA + 2,4-D, respectively. Lashin and Mamdouh (2014) reported in Cucumis sativus that the maximum weights of calli (4.67 g/Jar) were obtained from internode segments cultured on MS media containing 1 mg/l of NAA + 1 mg/l of 2,4-D. Similar outcomes were attained by Ewais (1995), who used kinetin and 2,4-D to produce cucumber calluses in large quantities. A fresh weight (0.428 g/explant) of callus in Grewia tenax after 28 days of culture was obtained. The fresh biomass increased up to 13 g when the callus was subcultured on 2.0 mg/l of NAA + 1.5 mg/l of BA-containing medium (Daffalla et al., 2019).

Beigmohamidi et al. (2021) induced callus from internodes of *Plumbago europaea* L., and maximum shoot induction was observed at MS medium containing 2 mg/l of BA and 0.1 mg/l of NAA. Tiwari et al. (1998) reported that, in *Bacopa monnieri* plants, internodal segments inoculated on MS medium containing 1.5–2.0 mg/l of BA produced the highest number of adventitious shoot buds. Maximum root induction was observed at 1.0 mg/l of IAA when internodal segments were used as explants. The use of NAA (0.5–2 mg/l) resulted in efficient rooting compared to the control. These results agree with a study by Rasheed et al. (2013) on watermelon, where it was reported that the maximum number of roots was seen in MS medium containing 0.1 mg/l of NAA. The present study indicated a maximum root number in MS medium containing 0.50 mg/l of NAA. Maximum root length was observed in MS medium containing 1.0 mg/l of IAA. A maximum number of roots and root length were observed in MS medium with 1.0 mg/l of NAA in the *B. monnieri* plant by Tiwari et al. (1998).

## Conclusion

Germination, induction, and morphological characteristics are important parameters widely used to study the effects of essential and nonessential compounds on plants during *in vitro* studies. Different parameters like callus induction, multiplication, growth of root and shoot systems, and biomass production were studied to explore the potential of explants (internodal segment, leaves, and nodal segments) for callus induction and plantlet regeneration in *S. lycopersicum* L. under treatment with different plant growth regulators.

According to the current investigation, 2,4-dichlorophenoxyacetic acid (2,4-D) at a dosage of 0.25 mg/l produced the highest percentage of callus induction and proliferation among the various PGRs. The best plant growth regulator for shooting was 0.75 mg/l of BA, and for rooting, it was 1.0 mg/l of IAA. The best response in terms of all growth parameters and regeneration in *S. lycopersicum* was obtained when nodal segments were used as explants, followed by internodal segments and leaves.

The establishment of regeneration studies in plants is a prerequisite for genetic transformation. The results of the present work can be used as a basis for the efficient transmission of desired traits through genetic transformation procedures. The findings of this study are recommended for the establishment of *in vitro* protocols for future studies to be conducted on *S. lycopersicum* L.
